# Microglia associated research in depression: an exploratory trends analysis

**DOI:** 10.3389/fnagi.2026.1757943

**Published:** 2026-03-11

**Authors:** Yan-Jun Chen, Qin-Quan Zhu, Ming-Rong Xie, Di Zhang, Yun-Fei Shuai, Ying Li, Nan Zhang, Wei-Xing He, Yan-Hong Liao, Qing-Jia Zeng, Zhuo Liu, Sheng-Qiang Zhou, Bo Li

**Affiliations:** 1 The First Hospital of Hunan University of Chinese Medicine, Changsha, China; 2 Graduate School of Hunan University of Chinese Medicine, Changsha, China; 3 The First Clinical College of Nanjing University of Chinese Medicine, Nanjing, China; 4 National TCM Master Liu Zuyi Inheritance Studio, Hunan Provincial Hospital of Integrated Traditional Chinese and Western Medicine (The Affiliated Hospital of Hunan Academy of Traditional Chinese Medicine), Changsha, China

**Keywords:** depression, gut microbiota, kynurenine pathway, microglia, neuroinflammation

## Abstract

**Background:**

Depression is a common and highly debilitating severe affective disorder. Relevant studies have indicated that microglia are closely associated with the progression of depression. This study conducts a bibliometric analysis on the research related to microglia in depression, aiming to clarify the evolution process, research hotspots, and future research directions in this field.

**Method:**

Relevant publications were retrieved from the Scopus and Web of Science databases. The software tools CiteSpace, VOSviewer, and Bibliometrix were employed for visual analysis.

**Results:**

A total of 2,305 publications on microglia in depression were included in the analysis. From 2001 to 2024, research in this area generally exhibited an upward trend. China had the largest number of research outcomes. Nantong University had the highest research output. *Brain, Behavior, and Immunity* was the journal with the largest number of relevant publications. Dr. Huang Chao was the most prolific author. High-frequency keywords included depression, microglia, neuroinflammation, inflammation, hippocampus, metabolism, cytokines, IL-1β, lipopolysaccharide, stress, and anxiety. Kynurenine pathway, NF-κB signaling, hypothalamus hypophysis adrenal system, and gut microbiota have emerged as active research topics in recent years.

**Conclusion:**

This study provides a bibliometric perspective on microglia in depression. Research institutions from various countries have collaborated to advance this field. The neuroinflammation triggered by microglia has become an important theoretical framework for understanding the biological mechanisms of depression. Kynurenine pathway, NF-κB signaling, hypothalamus hypophysis adrenal system, and the gut microbiota may be the directions of future research.

## 1 Introduction

Depression is a prevalent and severely debilitating affective disorder. Its core clinical manifestations include persistent low mood, reduced interest, and decreased energy (Malhi and Mann, 2018). The pathogenesis of depression has traditionally been attributed to dysfunction in monoamine neurotransmitters, including serotonin, norepinephrine, and dopamine, which constitutes the pharmacological basis for most first-line antidepressants (Belmaker and Agam, 2008). However, the monoamine hypothesis does not fully account for the entirety of depression’s pathophysiology, and a substantial proportion of patients exhibit poor responses to existing therapies (Al-Harbi, 2012). This underscores the urgent need to explore alternative pathogenic mechanisms and identify novel therapeutic targets.

Microglia, the resident immune cells of the central nervous system (CNS), continuously survey the microenvironment by extending and retracting their processes under physiological conditions. They perform crucial functions, including clearing apoptotic cellular debris, synaptic pruning, and maintaining CNS homeostasis (Augusto-Oliveira et al., 2025). Upon pathological stimulation, microglia rapidly activate and polarize into distinct functional phenotypes. The classically activated M1 phenotype predominantly releases pro-inflammatory cytokines (such as IL-1β, IL-6, and TNF-α), which can exacerbate neuroinflammation and neuronal injury (Tang and Le, 2016). In contrast, the alternatively activated M2 phenotype primarily secretes anti-inflammatory factors (such as IL-10 and TGF-β) that promote tissue repair and inflammation resolution (Cherry et al., 2014). Chronically overactivated microglia can drive persistent neuroinflammation, directly or indirectly damaging neurons via excessive production of inflammatory mediators and reactive oxygen species, thereby playing a central role in the pathogenesis of various neurological disorders (Li and Barres, 2018).

Accumulating evidence indicates a close association between microglia and the progression of depression. Activated microglia, particularly those polarized towards the M1 phenotype, release pro-inflammatory cytokines that may compromise the blood–brain barrier (BBB) and amplify neuroinflammatory responses (Guo et al., 2022). These inflammatory mediators can disrupt neurotransmitter metabolism, suppress the expression of brain-derived neurotrophic factor, impair neurogenesis in emotion-related brain regions (e.g., the hippocampus), and detrimentally affect synaptic plasticity (Miller et al., 2009; Yirmiya et al., 2015). Consistent with this, clinical neuroimaging studies have revealed significant microglial activation in multiple brain regions of depression patients (Setiawan et al., 2015). Therefore, regulating the neuroinflammatory response through microglia may be an effective strategy for treating depression.

Bibliometrics is an interdisciplinary research field that employs mathematical and statistical methods to quantitatively analyze scholarly literature (Ninkov et al., 2022). Applying bibliometric approaches to the study of microglia in depression can objectively delineate the evolution, intellectual structure, research hotspots, and emerging frontiers within this field, thereby providing researchers with a comprehensive overview of the research landscape.

## 2 Methods

### 2.1 Data search

**Figure 1:**
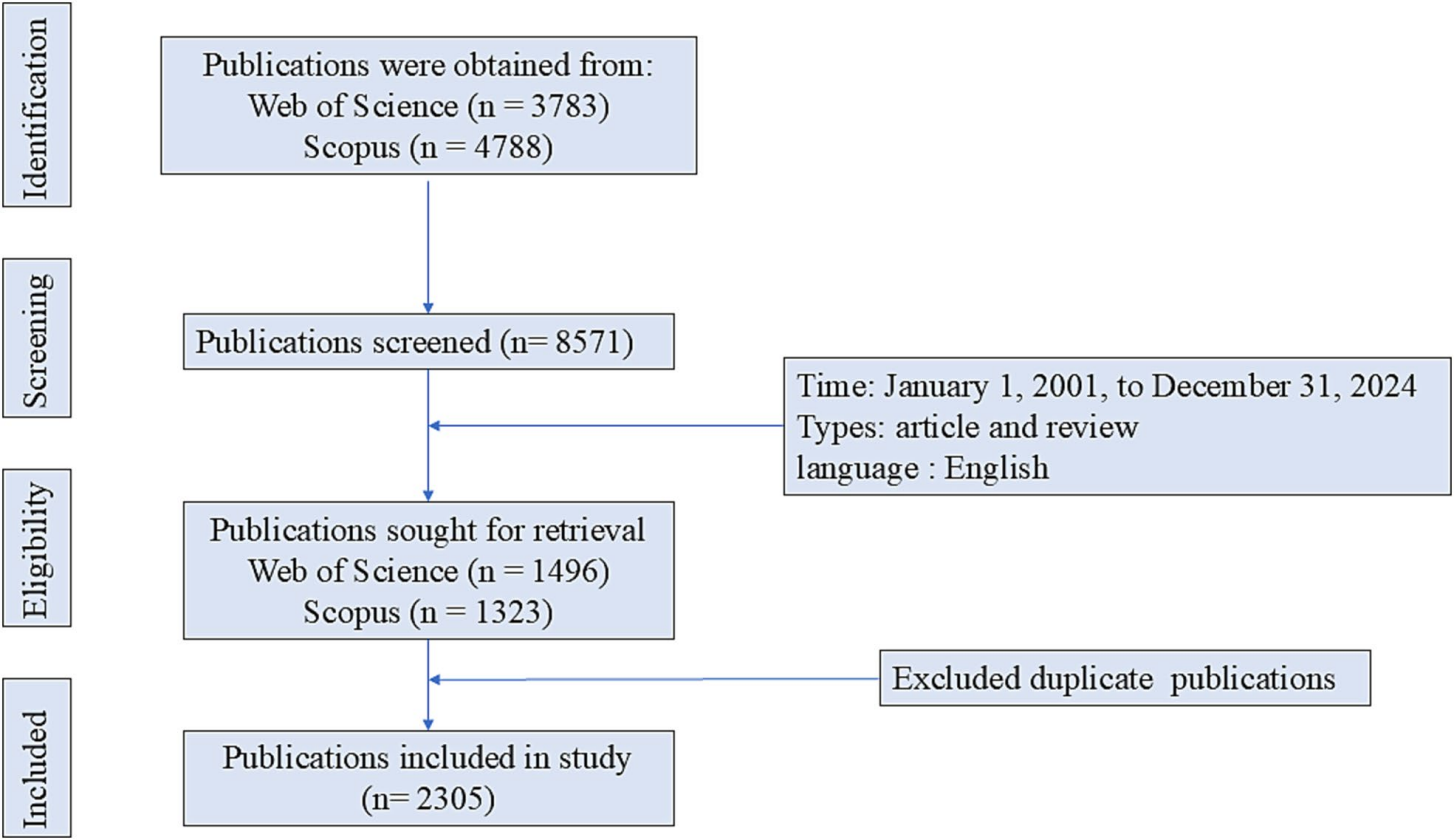
Flowchart of publication search process.

Publications related to microglia and depression were retrieved from the Scopus and Web of Science (WoS) databases. The WoS search formula was: (((((((((((((((((TS = (depressive disorder)) OR TS = (major depressive disorder)) OR TS = (major depression)) OR TS = (MDD)) OR TS = (depressions)) OR TS = (depression)) OR TS = (depressive)) OR TS = (depressed)) OR TS = (despondent)) OR TS = (depressive symptom)) OR TS = (depressive neuroses)) OR TS = (depressive syndrome)) OR TS = (recurrent depressive disorder)) OR TS = (single episode depressive disorder)) OR TS = (depressively)) OR TS = (depressiveness)) OR TS = (depressives)) AND ((((TS = (microglias) OR TS = (microglial cell)) OR TS = (microglial cells)) OR TS = (microglial)) OR TS = (microglia)). The Scopus search formula was: (((((((((((((((((TITLE-ABS-KEY(depressive disorder)) OR TITLE-ABS-KEY(major depressive disorder)) OR TITLE-ABS-KEY(major depression)) OR TITLE-ABS-KEY(MDD)) OR TITLE-ABS-KEY(depressions)) OR TITLE-ABS-KEY(depression)) OR TITLE-ABS-KEY(depressive)) OR TITLE-ABS-KEY(depressed)) OR TITLE-ABS-KEY(despondent)) OR TITLE-ABS-KEY(depressive symptom)) OR TITLE-ABS-KEY(depressive neuroses)) OR TITLE-ABS-KEY(depressive syndrome)) OR TITLE-ABS-KEY(recurrent depressive disorder)) OR TITLE-ABS-KEY(single episode depressive disorder)) OR TITLE-ABS-KEY(depressively)) OR TITLE-ABS-KEY(depressiveness)) OR TITLE-ABS-KEY(depressives)) AND ((((TITLE-ABS-KEY(Microglias) OR TITLE-ABS-KEY(microglial cell)) OR TITLE-ABS-KEY(microglial cells)) OR TITLE-ABS-KEY(microglial)) OR TITLE-ABS-KEY(microglia)). The inclusion criteria were as follows: (i) publication date between January 1, 2001, and December 31, 2024; (ii) document types limited to reviews and articles; (iii) publication language restricted to English. The exclusion criteria were: (i) publications irrelevant to the research topic; (ii) duplicate publications. The bibliographic records exported from the WoS and Scopus databases were first matched and merged using unique identifiers (DOI) and bibliographic details (title, authors, journal, year). Duplicate records were removed using the reference management software EndNote. Two investigators independently verified all records that were flagged as potential duplicates or whose matching status was uncertain. Verification was performed through direct comparison of abstracts, author lists, journal volume, issue, and page numbers. This procedure ensured that each entry in the final dataset represented a distinct published paper. Two researchers independently collected relevant literature based on the above inclusion criteria (Kappa = 0.99) and discussed the discrepant literature together. For a small number of disagreements that emerged during the screening process (21 papers in total), the two researchers reached consensus through joint discussion and reference to the original content. If there was still disagreement after the discussion, the third senior researcher would make the final decision as an arbitrator. After this process, a total of 2,305 publications were included for final analysis (Figure 1).

### 2.2 Data analysis

The data were analyzed using software CiteSpace, VOSviewer, and Bibliometrix, which were in line with the previous research methods (Chen et al., 2025a; Chen et al., 2025b). CiteSpace excels at tracing the historical progression and pivotal shifts within scholarly domains, effectively mapping the developmental pathways and intellectual transitions of research themes over time (Chen, 2004). VOSviewer specializes in constructing interactive relationship maps for collaborative and co-occurrence analyses, where its density visualization modes distinctly emphasize central research themes (van Eck and Waltman, 2010). Bibliometrix provides a streamlined solution for consolidating and examining bibliographic datasets, enabling the systematic detection of partnership patterns and emerging interdisciplinary themes, which collectively elevate the productivity of literature-based studies (Aria and Cuccurullo, 2017). The time range was set from January 2001 to December 2024, with each year as a slice, and the rest were set to default values. In the detection of burst keywords, the *γ* value was set to 1.0. The clustering algorithm based on modularity standardizes the data using association strength. Bradford’s Law was embedded within the R package of Bibliometrix.

## 3 Results

### 3.1 Publication trends

**Figure 2:**
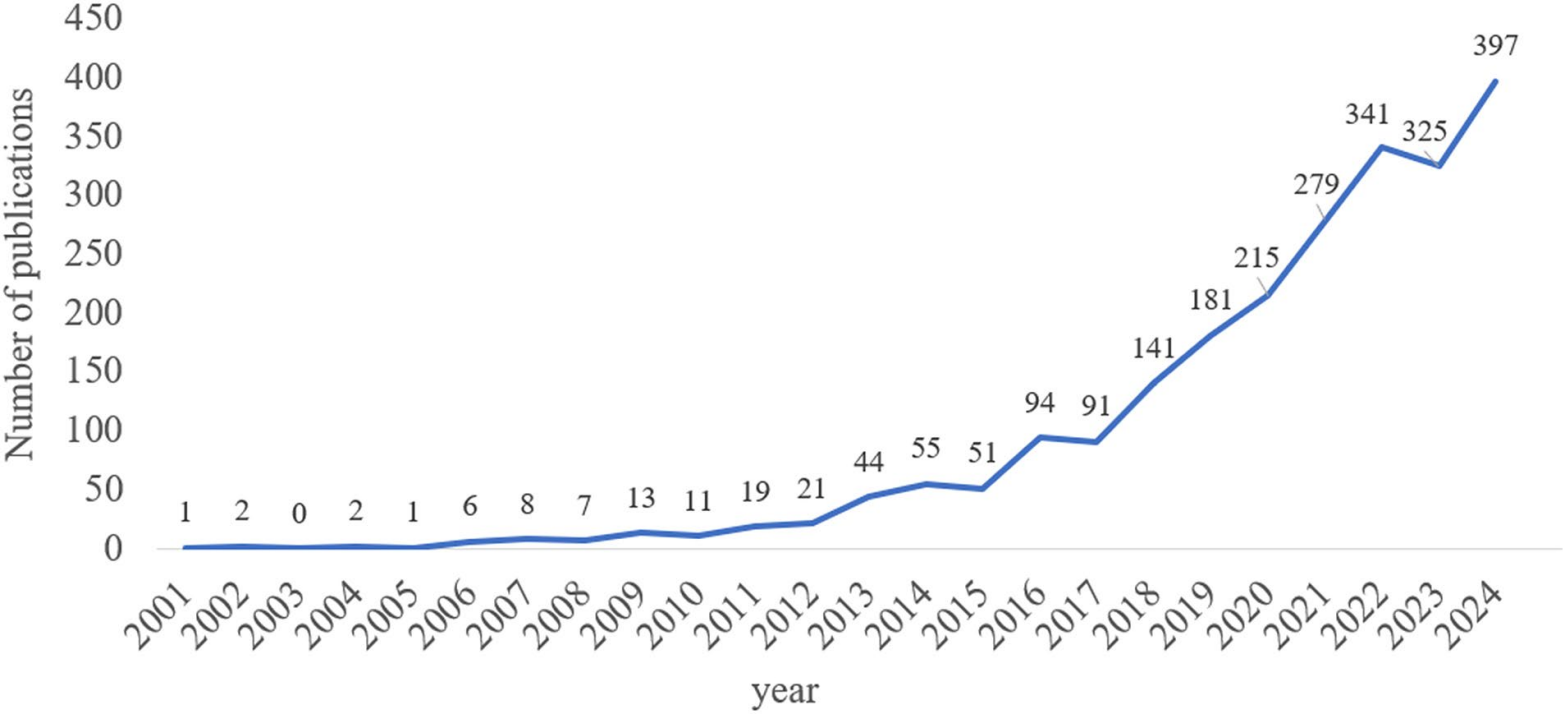
Annual number of publications on microglia and depression.

From 2001 to 2024, research on microglia in depression generally exhibited an upward trend. Annual publications were divided into three phases. The first phase (2001–2012) was characterized by a relatively small number of publications, reflecting the early stage of research with limited attention. The second phase (2013–2017) witnessed a steady growth in the number of publications, indicating a gradual rise in research interest. The third phase (2018–2024) experienced a rapid increase in publications, peaking in 2024 (397 publications) (Figure 2).

### 3.2 Country

**Figure 3:**
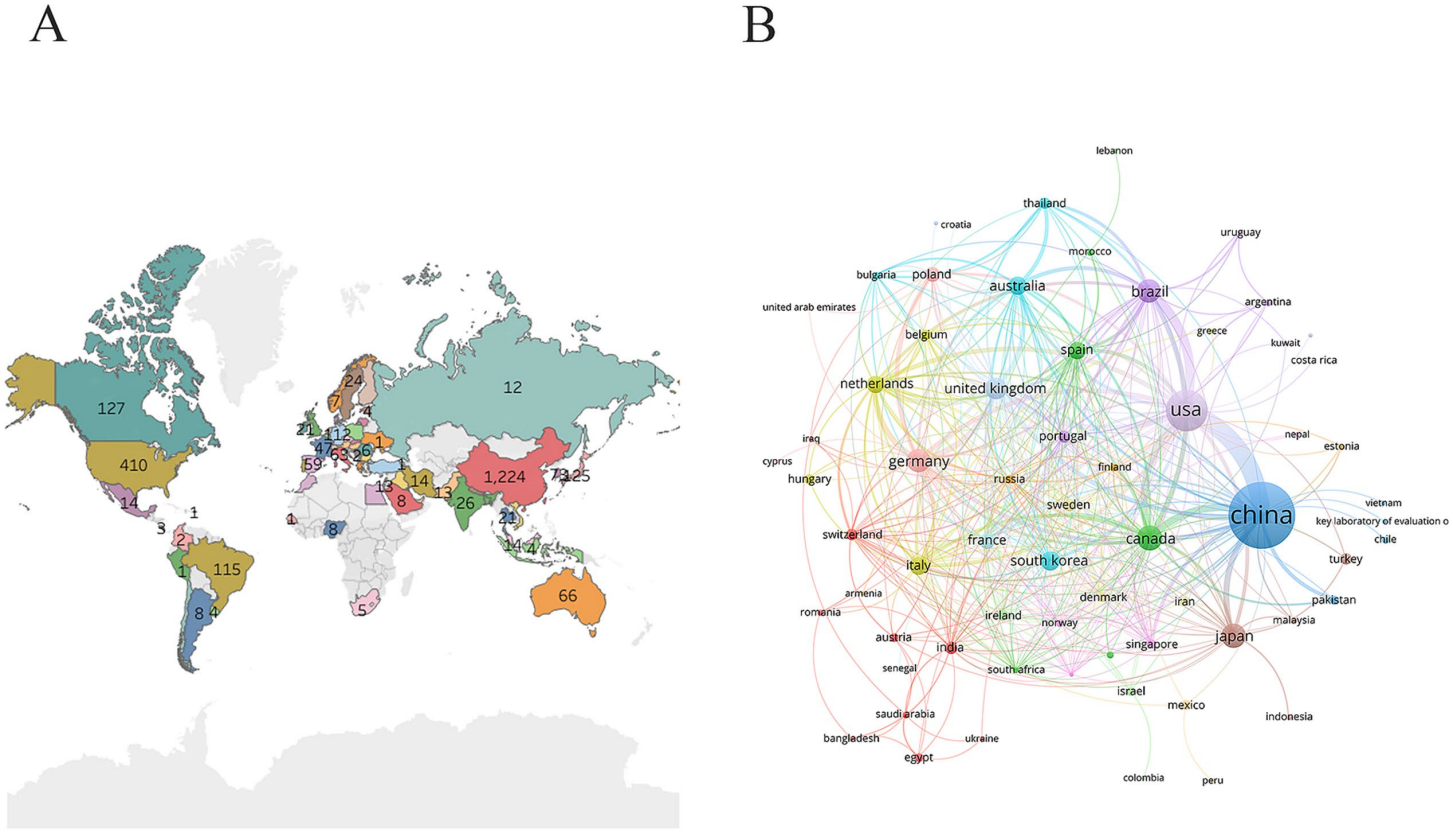
Country analysis. **(A)** Geographic distribution of publications. **(B)** International collaborative network.

Global research on microglia and depression was primarily concentrated in Asia and North America (Figure 3A). China had the largest number of research outcomes (1,224 publications) and ranked first. Although the United States, Canada, Germany, and Australia had fewer publications than China, their average citation count exceeds 60, underscoring the high quality of their research output (Table 1). Brazil ranked among the top five in terms of output with 115 publications, highlighting the active involvement and growing influence of Latin American countries in this cutting-edge area of neuroscience. Collaborative efforts involved countries across multiple continents, with the closest collaboration observed between China and the United States (Figure 3B).

**Table 1:** The top 10 countries.

Rank	Country	Number of publications	Citations	Average citation
1	China	1,224	33,093	27.04
2	USA	410	25,049	61.10
3	Canada	127	8,204	64.60
4	Japan	125	5,008	40.06
5	Brazil	115	4,485	39.00
6	Germany	112	7,371	65.81
7	United Kingdom	92	5,299	57.60
8	South Korea	73	2,626	35.97
9	Australia	66	4,869	73.77
10	Italy	63	2,629	41.73

### 3.3 Institutions

**Figure 4:**
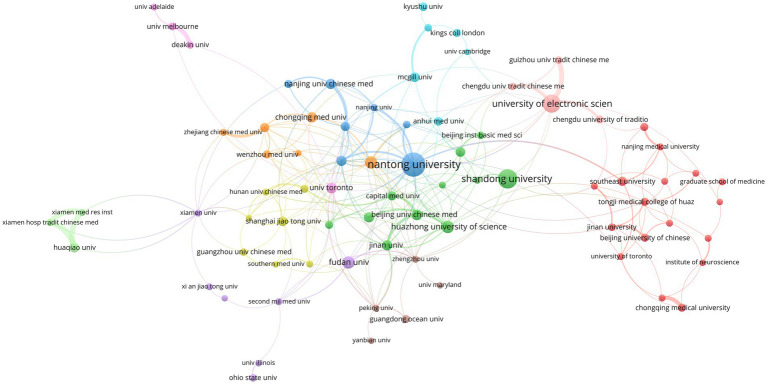
Institutional collaborative network.

Collaboration patterns among institutions reflected both international and domestic partnerships (Figure 4). Among the top 10 institutions, nine were from China, and the only international institution was the University of Toronto (Table 2). This aligned with China’s leading position in total national publication output and illustrated the high concentration of scientific research capabilities within its top universities and research institutes. Nantong University had the highest number of publications (45), indicating the highest research output. Although the University of Toronto ranked seventh with 21 publications, it had the highest total citations (2,878) and a considerably higher average citation rate (137.05) than other institutions, reflecting its high research quality, significant academic impact, and broad recognition.

**Table 2:** The top 10 institutions.

Rank	Institution	Documents	Citations	Average number of citations
1	Nantong University	65	1,415	21.77
2	Shandong University	48	1,384	28.83
3	University of Electronic Science and Technology of China	44	2,334	53.05
4	Huazhong University of Science and Technology	26	1,080	41.54
5	Fudan University	25	1,499	59.96
5	Chinese Academy of Sciences	25	1,026	41.04
7	University of Toronto	21	2,878	137.05
7	Beijing University of Chinese Medicine	21	556	26.48
8	Chongqing Medical University	20	833	41.65
8	Southeast University	20	861	43.05
8	Wuhan University	20	316	15.80

### 3.4 Journals

**Figure 5:**
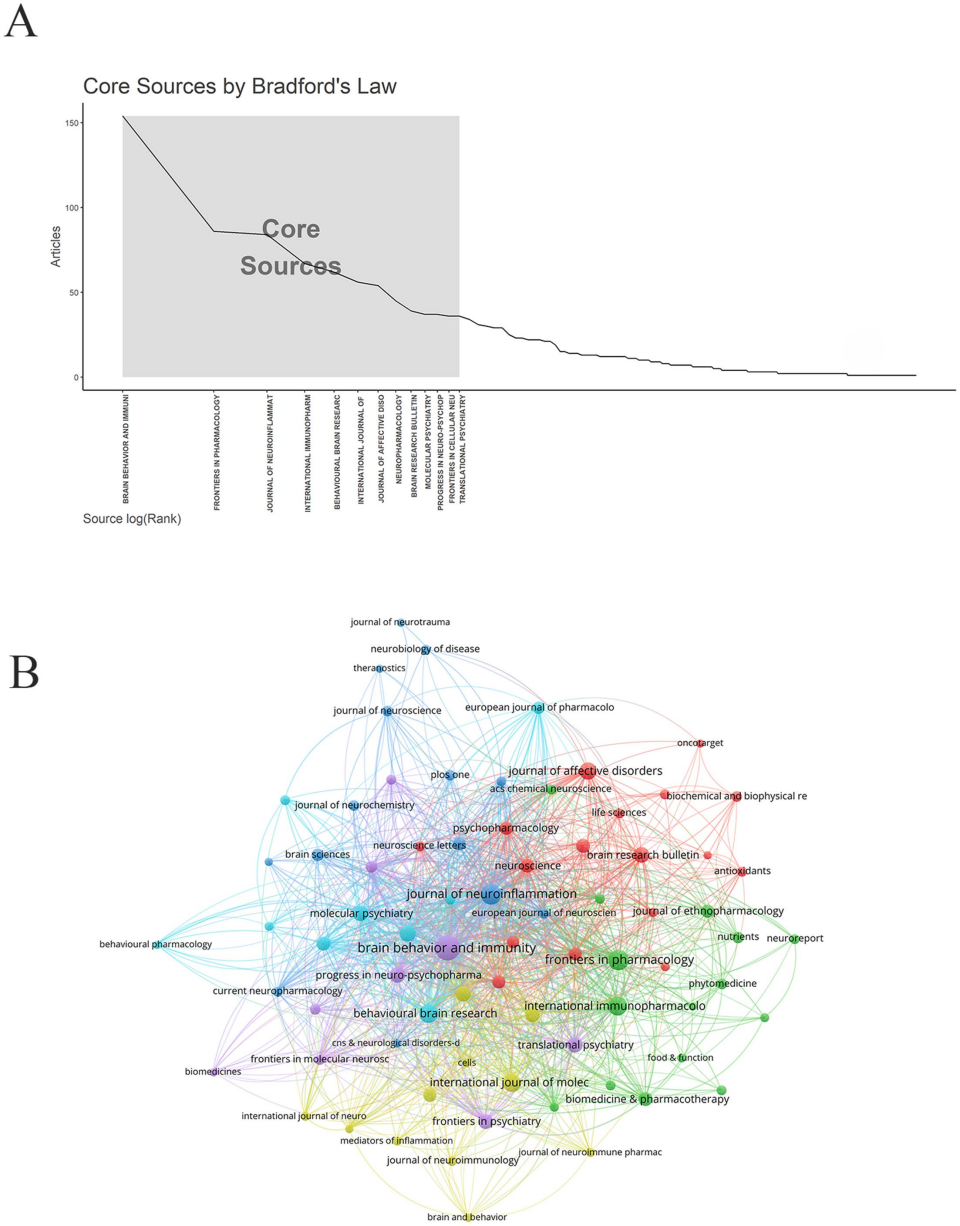
Journal analysis. **(A)** Core journal. **(B)** Journal network.

Based on Bradford’s Law, 13 core journals were identified (Figure 5A). Collectively, these journals represented a highly concentrated knowledge base in microglia-related depression research, spanning the interdisciplinary frontiers of neuroscience, immunology, and psychiatry. *Brain, Behavior, and Immunity* served as a cornerstone in the field, publishing the largest number of publications (154) while maintaining a high average citation rate (47.64) and an impact factor of 7.6, reflecting its consistent output of high-quality research. *The Journal of Neuroinflammation* and *Molecular Psychiatry* were regarded as benchmarks in the field, with a relatively high impact factor (IF = 10.1) and an average citation rate (95.70 and 79.76, respectively). This suggested that the studies published in these two journals are groundbreaking and represent key advances driving progress in the field (Table 3; Figure 5B).

**Table 3:** The top 10 journals.

Rank	Source	Documents	Citations	Average citations	IF	JCR
1	Brain Behavior and Immunity	154	7,337	47.64	7.6	Q1
2	Frontiers in Pharmacology	86	2,482	28.86	4.8	Q1
3	Journal of Neuroinflammation	84	8,039	95.70	10.1	Q1
4	International Immunopharmacology	67	915	13.66	4.7	Q2
5	Behavioural Brain Research	62	1,549	24.98	2.3	Q2
6	International Journal of Molecular Sciences	56	1,543	27.55	4.9	Q1
7	Journal of Affective Disorders	54	730	13.52	4.9	Q1
8	Neuropharmacology	45	1,002	22.27	4.6	Q1
9	Brain Research Bulletin	39	762	19.54	3.7	Q2
10	Molecular Psychiatry	37	2,951	79.76	10.1	Q1
10	Progress in Neuro-Psychopharmacology & Biological Psychiatry	37	2,155	58.24	3.9	Q1

### 3.5 Authors

**Figure 6:**
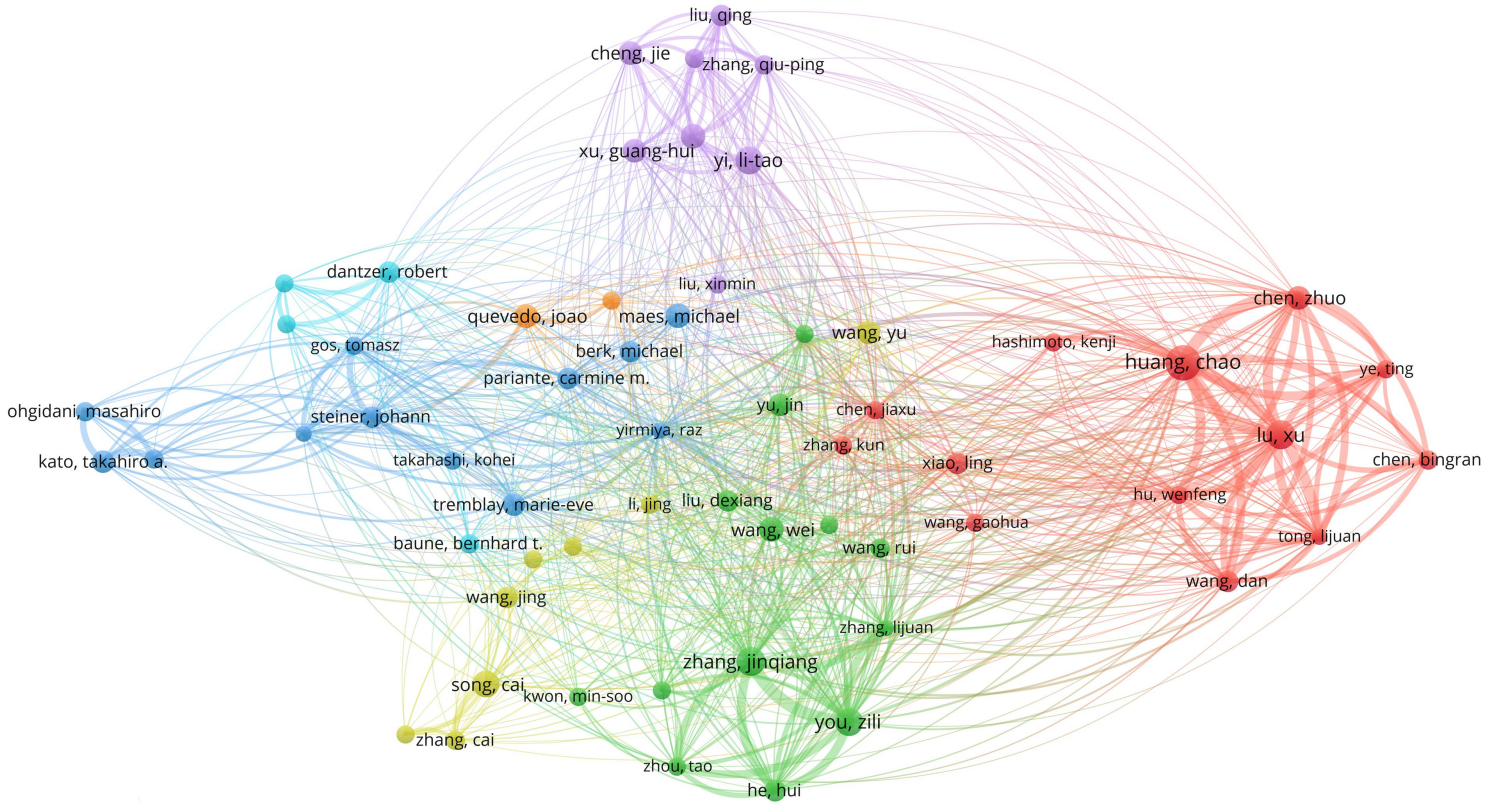
Author collaboration network.

In studies related to microglia in depression, Chinese scholars have maintained a dominant position (Figure 6), reflecting China’s strong scientific research activity and resource advantages in this area. Dr. Huang Chao led with 22 publications, establishing him as a core researcher. Dr. Lu Xu and Dr. Zhang Jinqiang tied for second place, each with 16 publications (Table 4). Multiple authors published between 11 and 15 articles, indicating the emergence of a stable and highly productive group of scholars in this research domain.

**Table 4:** The top 10 authors.

Rank	Author	Documents	Country	Institution
1	Dr. Huang, Chao	22	Nantong University	China
2	Dr. Lu, Xu	16	Nantong University	China
2	Dr. Zhang, Jinqiang	16	Guizhou University of Traditional Chinese Medicine	China
4	Dr. Yi, Litao	15	Huaqiao University	China
4	Dr. You, Zili	15	University of Electronic Science and Technology of China	China
6	Dr. Song, Cai	14	Guangdong Ocean University	China
7	Dr. Li, Chengfu	12	Xiamen Hospital of Traditional Chinese Medicine	China
7	Dr. Michael Maes	12	Deakin University	Australia
7	Dr. Wang, Wei	12	Second Military Medical University	China
10	Dr. Chen, Zhuo	11	Nantong University	China
10	Dr. Cheng, Jie	11	Huaqiao University	China
10	Dr. Joao Quevedo	11	University of Texas Health Science Center at Houston	USA
10	Dr. Wang, Yu	11	China Academy of Chinese Medical Sciences	China
10	Dr. Xu, Guanghui	11	Xiamen Medicine Research Institute	China

### 3.6 Keywords

**Figure 7:**
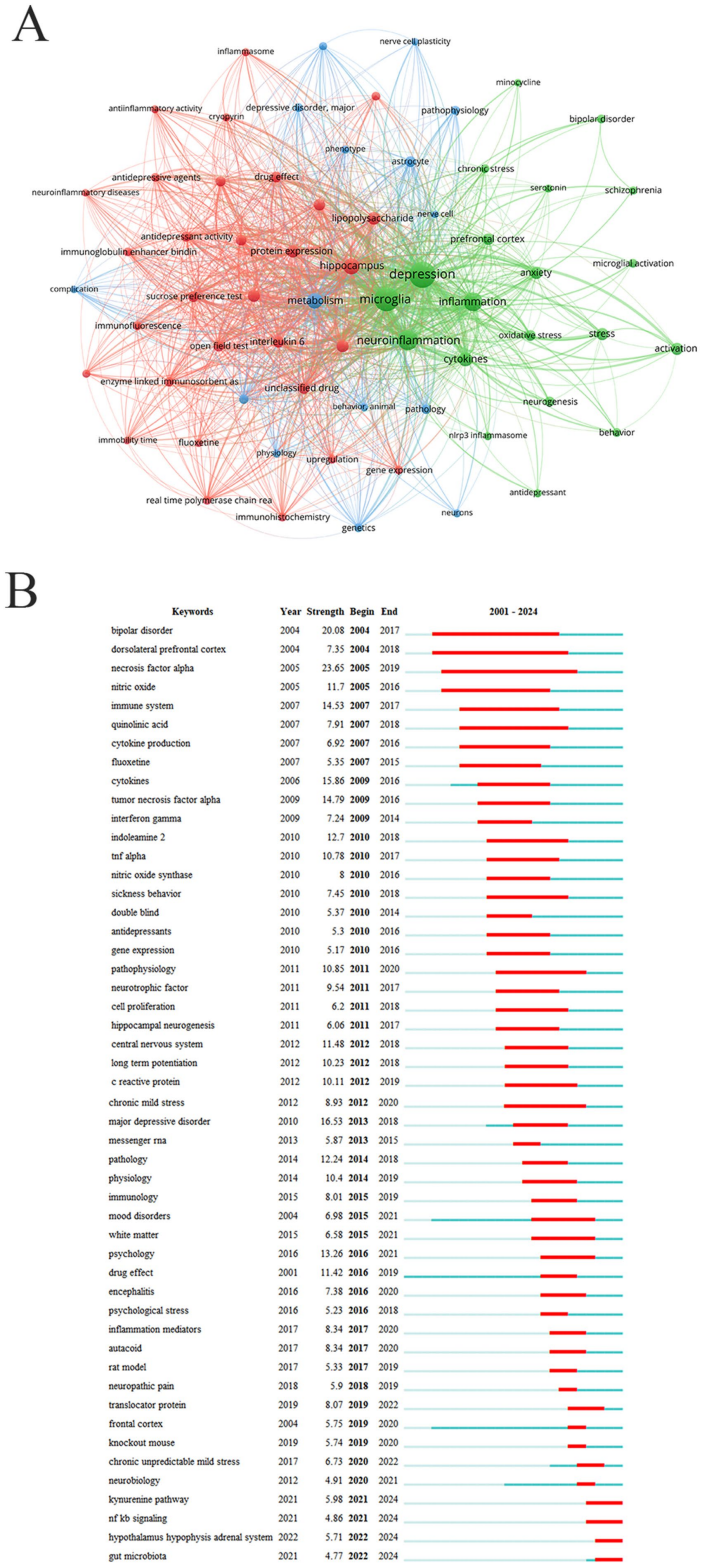
Keywords analysis. **(A)** Keywords network. **(B)** Keywords with the strongest citation bursts.

Keywords represent focal points of research within a given field. By analyzing and categorizing frequently used keywords, researchers can quickly identify current core research topics. High-frequency keywords in this study included depression (1,695 times), microglia (1,385 times), neuroinflammation (886 times), inflammation (744 times), hippocampus (543 times), metabolism (465 times), cytokines (408 times), interleukin 1 beta (IL-1β) (301 times), lipopolysaccharide (298 times), stress (288 times), anxiety (286 times) (Figure 7A). Burst keyword analysis is commonly used to detect terms that experience a sudden increase in research interest over a specific period, reflecting emerging trends and key areas in the study of microglia in depression. Research focus exhibits a clear temporal evolution, which can be roughly divided into three stages (Figure 7B). From 2004 to 2010, studies primarily concentrated on inflammation and immune system activation. Keywords such as “tumor necrosis factor-alpha” and “cytokines” marked the initial establishment of the inflammatory hypothesis of depression. During the period from 2011 to 2017, the focus shifted toward the interaction between immunity and neuroplasticity. The emergence of burst keywords like “neurotrophic factor” and “hippocampal neurogenesis” reflected growing attention to microglia-mediated regulation of synaptic function and neurogenesis. Since 2018, research has further integrated multi-system regulatory networks, as indicated by keywords such as “kynurenine pathway,” “gut microbiota,” and “NF-κB signaling.” This trend signifies that the field has entered a stage of systematic neuroimmunology and translational exploration.

## 4 Discussion

### 4.1 General information

A total of 2,305 studies on microglia in depression were included in the analysis. From 2001 to 2024, research in this area generally exhibited an upward trend. Although China had the highest total number of publications, the average number of citations per paper in countries such as the United States, Canada, Germany, and Australia was significantly higher. Nantong University had the highest research output. *Brain, Behavior, and Immunity* was the journal with the largest number of relevant publications. Dr. Huang Chao was the most prolific author.

### 4.2 Hotspots and frontiers

High-frequency keywords included depression, microglia, neuroinflammation, inflammation, hippocampus, metabolism, cytokines, IL-1β, lipopolysaccharide, stress, and anxiety. These keywords clearly reflect one of the most important and cutting-edge areas in current depression research: the neuroinflammation hypothesis. Dysfunction of microglia has become a central link connecting immune abnormalities and neuropsychiatric disorders. The mechanisms by which microglia promote depression are multifaceted. Under pathological conditions such as stress, microglia become overactivated, transitioning from a resting state to a “pro-inflammatory phenotype” (M1 type) and releasing large amounts of pro-inflammatory cytokines such as IL-1β, IL-6, and TNF-α. These inflammatory factors not only directly impair neuronal function but also, by activating the indoleamine 2,3-dioxygenase (IDO) pathway, shift tryptophan metabolism toward the kynurenine pathway. This leads to increased production of neurotoxic quinolinic acid and reduced synthesis of 5-hydroxytryptamine, thereby exacerbating monoamine system dysfunction and mediating depressive-like behaviors (Dantzer et al., 2008; Stein et al., 2017). Microglial dysfunction profoundly affects synaptic plasticity and neural networks. Under normal physiological conditions, microglia prune redundant or damaged synapses, maintaining the precision of neural circuits (Tremblay et al., 2011). However, in chronic stress or inflammatory states, their phagocytic function becomes dysregulated, which may result in excessive and non-selective synaptic engulfment. This is particularly prominent in brain regions closely related to emotional regulation, such as the prefrontal cortex and hippocampus (McEwen et al., 2016; Schafer et al., 2012). Such loss of synaptic connections is considered closely associated with the cognitive symptoms and emotional processing deficits in depression (Paolicelli et al., 2011). Additionally, abnormal microglial activation inhibits neurogenesis in regions such as the hippocampus (Musaelyan et al., 2014). Brain-derived neurotrophic factor (BDNF) is crucial for neuronal survival and differentiation, and disruption of BDNF signaling is a well-established pathological mechanism in depression (Duman and Monteggia, 2006). Inflammatory factors released by microglia suppress BDNF expression, disturbing the balance between neurotrophic support and inflammatory signals. This leads to reduced neurogenesis and weakened emotional buffering and cognitive functions of the hippocampus (Slavich and Irwin, 2014; Cacci et al., 2005). Ultimately, core depressive symptoms driven by hippocampal dysfunction—such as low mood, anhedonia, and feelings of hopelessness—are often accompanied by anxiety. Microglia are not passive observers in depression but active regulators at the neuro-immune interface. Their abnormal activation, through multiple parallel and interacting pathways—including the release of inflammatory mediators, disruption of synaptic homeostasis, and impairment of neurotrophic support—collectively constitutes the neuroimmune pathological basis of depression. It is noteworthy that the role of microglia is dual. Under certain conditions, microglia can switch to an “anti-inflammatory phenotype” (M2 type), exerting tissue-repair and anti-inflammatory effects. Therefore, emerging therapeutic strategies for depression do not aim simply to inhibit microglia, but rather to restore their dynamic balance and shift them from a harmful pro-inflammatory state toward a protective, homeostasis-promoting phenotype.

Analyzing the temporal sequence of burst keywords can reveal the evolving research paradigm regarding microglia in depression, which parallels developments in neuroimmunology and psychiatry. Early studies (2004–2010) established the foundational role of inflammation in depression pathology. Mid-period research (2011–2017) deepened the understanding of microglia as a central immune hub, elucidating their involvement in disease processes through mechanisms such as neural plasticity and neurogenesis, thereby advancing the field from correlation to causation and mechanism. Recent work (2018–2024) exhibits a clear trend toward systematic and integrative approaches, focusing on multidimensional regulatory networks including the gut–brain axis, metabolic pathways, and neuroendocrine systems. The kynurenine pathway, NF-κB signaling, the hypothalamic–pituitary–adrenal axis, and gut microbiota have emerged as particularly active research topics in recent years and may represent future cutting-edge directions. In the pathophysiology of depression, microglial activation drives an imbalance in the kynurenine pathway, ultimately leading to neurotoxicity and impaired neuroplasticity, thereby contributing to depressive symptoms (Dantzer, 2017). Pro-inflammatory cytokines (especially IFN-γ and TNF-α) strongly induce IDO1 in microglia. IDO1 is the rate-limiting enzyme of the kynurenine pathway, and its activation shifts tryptophan metabolism away from serotonin synthesis toward the kynurenine pathway (Dantzer et al., 2008). Under the action of kynurenine 3-monooxygenase, kynurenine is metabolized into 3-hydroxykynurenine and quinolinic acid (Schwarcz and Stone, 2017). Quinolinic acid acts as a potent NMDA receptor agonist, inducing glutamate excitotoxicity and leading to neuronal damage and death (Guillemin, 2012). In depression, chronic stress or systemic inflammation persistently activates the NF-κB pathway within microglia, promoting excessive production of pro-inflammatory cytokines (such as IL-1β and IL-6) and thereby triggering neuroinflammation (Weber et al., 2017). NF-κB activation in microglia elevates inflammatory levels in key emotion-regulating brain regions, including the prefrontal cortex, hippocampus, and amygdala (Troubat et al., 2021). The suppression of hippocampal neurogenesis and synaptic loss in the prefrontal cortex are closely associated with depressive symptoms (such as anhedonia and cognitive impairment) (Duman and Monteggia, 2006; Christoffel et al., 2011). Studies suggest a vicious cycle in depression involving “stress–HPA axis activation–microglia–mediated neuroinflammation-depression” (Pariante, 2017). Chronic stress is a major trigger of depression, leading to HPA axis overactivation and elevated glucocorticoid levels (de Kloet et al., 2005). High glucocorticoid levels prime microglia, prompting them to release large quantities of pro-inflammatory cytokines (Frick et al., 2013). These cytokines further disrupt HPA axis balance and directly damage neurons, resulting in reduced neural plasticity and depressive behavioral symptoms (Miller and Raison, 2016). From the perspective of the gut-brain axis, the gut microbiota acts as a regulatory hub and signal transduction center in the interplay between microglia and depression. Dysbiosis can compromise the intestinal epithelial barrier, permitting bacterial lipopolysaccharides (LPS) and other microbial products to enter the systemic circulation. This triggers peripheral inflammatory responses and impairs blood–brain barrier integrity. LPS and inflammatory factors serve as potent activators of microglia, either entering the brain directly or activating perivascular microglia, thereby driving neuroinflammation (D'Mello et al., 2015). Microbiota-derived metabolites, such as short-chain fatty acids (SCFAs), can cross the blood–brain barrier and modulate microglial function. Butyrate, a key SCFA, plays a crucial role in maintaining microglial homeostasis by inhibiting excessive activation, exerting anti-inflammatory effects, and promoting neuroprotection (Huuskonen et al., 2004). Through multiple direct and indirect pathways, the gut microbiota regulates the phenotype and function of microglia, influencing neuroinflammation and neuroplasticity, and ultimately affecting emotional behavior. These interactions form a “microbiota-immune-brain” pathophysiological circuit.

### 4.3 Clinical and drug research

Clinical studies have primarily investigated the role of microglia in depression using neuroimaging and post-mortem brain tissue analyses. Positron emission tomography has clearly demonstrated that the total distribution volume of the translocator protein (TSPO VT) is significantly increased in multiple brain regions, including the prefrontal cortex, anterior cingulate cortex, and insula, in patients with depression (Setiawan et al., 2015). Moreover, the increase in TSPO VT in the anterior cingulate cortex was positively correlated with depression severity. These findings provide strong *in vivo* imaging evidence supporting the neuroinflammatory hypothesis of depression and suggest that therapies targeting microglial activation may have therapeutic potential. Immunohistochemical analysis of post-mortem brain tissue revealed that in patients with severe depression, the density of microglia expressing quinolinic acid (QUIN) was significantly increased in the subgenual anterior cingulate cortex and anterior midcingulate cortex, and these microglia exhibited morphological signs of activation (Steiner et al., 2011). This indicates that microglial activation in depression is region-specific and, through the production of the NMDA receptor agonist QUIN, links neuroimmune abnormalities with disruptions in glutamatergic neurotransmission. These observations provide direct histological support for the immune hypothesis of depression.

The mechanism of action of anti-inflammatory drugs in depression is primarily based on the inflammation hypothesis of depression. Clinical research has largely focused on non-steroidal anti-inflammatory drugs (NSAIDs) and cytokine inhibitors. NSAIDs, such as COX-2 inhibitors, reduce the synthesis of inflammatory mediators like prostaglandin by inhibiting cyclooxygenase. This leads to decreased levels of pro-inflammatory cytokines, which indirectly help restore the balance of tryptophan metabolism and enhance neurotrophic support (Müller, 2019). Clinical trials indicate that NSAIDs can moderately improve depression scores. Notably, the selective COX-2 inhibitor celecoxib has been shown to significantly alleviate depressive symptoms. When used short-term (6–12 weeks), it does not substantially increase the risk of adverse reactions such as gastrointestinal, cardiovascular, or infectious events (Köhler et al., 2014). Another type of research involves biological agents targeting specific cytokines. A systematic review and meta-analysis of trials in chronic inflammatory diseases indicated that anti-IL-6 antibodies, such as tocilizumab, produce a statistically significant improvement in depressive symptoms (Kappelmann et al., 2018). This antidepressant effect was positively correlated with the baseline severity of depressive symptoms. This suggests that cytokines (e.g., IL-6) may play a key role in the pathogenesis of depression. A randomized double-blind clinical trial explored the efficacy of the TNF-*α* inhibitor infliximab in treatment-resistant depression and analyzed the predictive value of baseline inflammatory markers (Raison et al., 2013). The results demonstrated that, although infliximab was not superior to placebo in the overall patient population, it significantly improved depressive symptoms and reduced inflammation levels in the high-inflammation subgroup (baseline hs-CRP > 5 mg/L). In contrast, the placebo showed better effects in patients with low inflammation. This finding indicates that inflammatory biomarkers may serve as predictive indicators for individualized treatment, suggesting that future anti-inflammatory therapies could be targeted to specific depression subtypes.

Currently, no drug has been officially approved by regulatory agencies specifically for targeting microglia in the treatment of depression. However, numerous existing and newly developed drugs have been found to exert antidepressant effects by modulating microglial activity. Certain conventional antidepressants, such as fluoxetine and sertraline, have been shown in preclinical models to inhibit M1 polarization of microglia, promote M2 polarization, and reduce the production of pro-inflammatory cytokines (Tynan et al., 2012). N-acetylcysteine, an antioxidant that modulates the glutamatergic system and neuroinflammation, has been shown in clinical studies to have adjunct efficacy in the treatment of depression (Berk et al., 2014); part of its mechanism involves inhibition of microglial activation and oxidative stress. Minocycline, a tetracycline antibiotic capable of crossing the blood–brain barrier, potently suppresses microglial activation. A clinical trial demonstrated that minocycline, when used as an augmenting agent in combination with standard antidepressants, improved symptoms in patients with treatment-resistant depression (Husain et al., 2017). Pioglitazone inhibits microglial activation by blocking the NF-κB pathway, and preliminary clinical studies suggest its potential utility in treating depression (Sepanjnia et al., 2012). Colony-stimulating factor 1 receptor inhibitors can suppress microglial activation and have exhibited antidepressant effects in preclinical models (Lei et al., 2020); however, their clinical application remains distant. Targeting microglia and neuroinflammation represents a promising new frontier in the treatment of depression and may offer new therapeutic options for the substantial number of patients who do not respond to existing therapies.

### 4.4 Challenge and opportunity

Under normal physiological conditions, microglia play crucial roles in synaptic pruning, circuit maturation, and the maintenance of brain homeostasis. Their activation also represents a protective response in early stages. However, suppression of all microglial activity may interfere with their normal physiological functions and pose long-term risks. A major challenge in drug development lies in how to precisely modulate their harmful, excessive activation while preserving their beneficial functions. The BBB presents a significant obstacle, and systemic immunosuppression may increase the risk of infection. Developing drugs with high central nervous system specificity and the ability to precisely regulate microglial functions remains technically challenging.

Microglia provide a new perspective in neuroimmunology, moving beyond the traditional monoamine neurotransmitter framework. Existing antidepressants are limited by their slow onset of action, notable side effects, and restricted efficacy. Targeting microglia is expected to become a new focus in drug development. Currently, the diagnosis of depression relies primarily on subjective symptom scales, lacking objective biomarkers. Positron emission tomography enables non-invasive detection of microglial activation levels *in vivo*, which may help identify depression patients with significant neuroinflammation and facilitate precise psychiatric diagnosis. Future research should focus on the following directions: (i) developing more specific microglia-targeted tracers and regulatory tools; (ii) conducting large-scale longitudinal clinical studies integrating multiple biomarkers (such as imaging, blood, and genetic) to deeply analyze the role of microglia across different stages and subtypes of depression; and (iii) exploring combination therapies, such as antidepressants together with anti-inflammatory agents.

## 5 Conclusion

This study provides a bibliometric perspective on microglia in depression. Research institutions from various countries have collaborated to promote the development of this field. The neuroinflammation triggered by microglia has become an important theoretical framework for understanding the biological mechanisms of depression. Kynurenine pathway, NF-κB signaling, hypothalamus hypophysis adrenal system, and the gut microbiota may be the directions of future research.

## Data Availability

The original contributions presented in the study are included in the article/supplementary material, further inquiries can be directed to the corresponding authors.
